# Analysis of neural coding of speech in monaural and binaural conditions in children with speech sound disorder

**DOI:** 10.1590/2317-1782/e20250421en

**Published:** 2026-07-10

**Authors:** Marciana da Costa Carlos, Thaís Nobre Uchôa Souza, Ranilde Cristiane Cavalcante Costa, Aline Tenório Lins Carnaúba

**Affiliations:** 1 Programa Associado de Pós-graduação em Fonoaudiologia, Universidade Estadual de Ciências da Saúde de Alagoas – UNCISAL - Maceió (AL), Brasil.; 2 Departamento de Fonoaudiologia, Universidade Estadual de Ciências da Saúde de Alagoas – UNCISAL - Maceió (AL), Brasil.

**Keywords:** Auditory Evoked Potential, Phonological Disorder, Children, Speech, Electrophysiology

## Abstract

**Purpose:**

To analyze whether children with phonological disorder present alterations in the neural coding of speech, using Frequency Following Response (FFR) under monaural and binaural conditions.

**Methods:**

This is an analytical, observational, cross-sectional study with a sample of 21 children with phonological disorders and 21 with typical development. They underwent the following procedures: medical history survey, phonological assessment, inspection of the external auditory canal, and audiological and electrophysiological evaluation, performed in one or two sessions. FFR was performed with the speech stimulus /da/, in two sweeps of 3000 stimuli at 80 dB HL, with a rate of 10.9 stimuli/second, a duration of 40 ms, and an analysis window of 74.677 ms.

**Results:**

Children with phonological disorders presented higher latency values ​​in all conditions than those with typical development, with statistical significance in the latency of wave V, and lower amplitudes than those with typical development, although without statistical significance. In terms of stimulus presentation, the binaural condition showed significantly larger amplitudes than the monaural conditions, suggesting the presence of a binaural summation effect.

**Conclusion:**

Children with phonological disorders present alterations in speech encoding, evidenced by delays in FFR responses under both monaural and binaural conditions.

## INTRODUCTION

The integrity of sensory pathways is essential for child development, since the processing of auditory information allows for the discrimination of acoustic cues important for phonological acquisition and the improvement of auditory skills. Thus, both the peripheral and central auditory pathways are fundamental for the acquisition of oral language^(1)^.

In this context, the maturation of the central auditory nervous system (CANS) plays a crucial role in the quality of auditory processing and, when impaired, can result in perceptual deficits and communication disorders^(1)^.

Language development, especially children's phonological skills, depends on the accurate perception of speech sounds. The refined processing of acoustic features, particularly the rapid temporal changes in speech dynamics, is essential for this development^(2)^. Therefore, when any of these processes related to the production and perception of sounds is disorganized, there is difficulty in acquiring the phonological system, which can lead to the omission or substitution of phonemes^(3)^, as observed in speech sound disorders (SSD).

SSD is a broad term that encompasses any difficulties related to the articulation or phonological representation of speech sounds, resulting in decreased intelligibility. Among the SSD subtypes, phonological disorder relates to alterations in the consolidation of the phonological system, characterized by recurrent phoneme substitutions and/or omissions, which generally affect a class of sounds and persist in age ranges where this would no longer be expected^(4)^.

Recent studies have shown that children with phonological disorder presented results below what is expected for their age in behavioral tests of central auditory processing^(5,6)^ and electrophysiological tests^(7-9)^. The latter includes the Frequency Following Response (FFR), which evaluates speech encoding through the synchronized activity of neurons in the auditory pathways, focusing on the brainstem, an area vital for language and hearing. Recent studies show that FFR can be a valuable tool for the differential diagnosis of disorders related to auditory processing, language, and speech^(8)^.

Due to the ability of the human auditory system to process differences in acoustic signals that reach one or both ears, FFR can be performed under monaural (right or left) and binaural (simultaneously in both ears) stimulation conditions. Monaural stimulation investigates the encoding of the speech stimulus in only one ear (enabling the analysis of auditory asymmetries or lateralization), while the binaural condition refers to the presentation of stimuli simultaneously in both ears (allowing for a more intense sound perception). The comparative analysis between these two conditions is important in evaluating the efficiency of isolated auditory encoding, as well as neural integration between the cerebral hemispheres, expanding the understanding of speech and language processing mechanisms^(10,11)^.

The measures extracted from the components analyzed in FFR also include latencies and amplitudes, which generate relevant data on the functioning of the auditory system. Latency is related to the time of the neural response to an auditory stimulus, which allows for the analysis of temporal synchronization during the neural encoding of a complex sound. Amplitude, in turn, indicates the robustness and strength of the electrical response generated in response to auditory stimuli, influenced by neural synchronization. Alterations in these measurements may indicate dysfunctions in temporal coding and integrity of the auditory pathways. Thus, the analysis of FFR latencies and amplitudes can contribute to the analysis of the neurobiological bases of speech and language disorders, bringing relevant implications for early diagnosis and the initiation of Speech-Language-Hearing (SLH) interventions^(12)^.

Unlike behavioral tests^(13)^, which depend on the child's response and concentration, the FFR offers an objective and precise measure of how the brain processes complex auditory sounds, such as speech^(14)^. It allows the identification of possible deficits in auditory perception, frequently present in children with phonological disorder, which involves difficulties in the perception and discrimination of distinctive features responsible for transmitting acoustic properties of speech sounds, such as frequency, voicing, and temporal patterns. Thus, FFR findings can help to understand auditory coding in phonological disorder and consequently assist in therapeutic strategies for reducing and eliminating this disorder^(2)^.

However, despite the increasing use of FFR in the investigation of language and speech disorders^(15)^, few studies have explored the differences between monaural and binaural stimulation in these populations, including children with phonological disorders. Therefore, considering that FFR allows for a detailed and objective analysis of the neural coding of speech sounds, and that children with phonological disorders have an impaired perception of language contrasts, this study aimed to analyze whether children with phonological disorders present alterations in the neural coding of speech, using FFR under monaural and binaural conditions.

## METHODS

This is an analytical, observational, cross-sectional study approved by the Research Ethics Committee (CEP) of the State University of Health Sciences of Alagoas (UNCISAL) with opinion number no. 6.692.677 and developed in UNCISAL’s Hearing and Technology Laboratory. All human research norms and guidelines were complied with, in accordance with Resolution 466/12 of the National Health Council of Brazil.

The sample consisted of two groups: Study Group (SG), formed by 21 children with phonological disorder, and Control Group (CG), composed of 21 typically developing children, matched according to sex and age. They were defined by comparing their mean values, resulting in 21 children per group. The parameters used to calculate the sample size were alpha = 0.05, beta = 0.1, standard deviation = 0.5, and difference between groups = 0.8, according to the results found by Gonçalves (2009)^(16)^.

The inclusion criteria for both SG and CG were pure-tone hearing thresholds within the normal range (up to 15 dB HL in octaves from 500 to 4000 Hz), normal visual inspection of the external auditory canal, type “A” tympanograms, and absolute and interpeak latencies referring to waves I, III, and V within the normal criteria bilaterally. The exclusion criteria for both groups were more than three ear infections in the current year, diagnosis of auditory neuropathy spectrum disorder (ANSD), genetic syndromes, neurological dysfunction, psychic or intellectual disability, and structural alterations in the speech organs that cause speech alterations (such as cleft lip and palate or short lingual frenulum).

The specific SG criterion was children with productive phonological processes in speech. The children included in the study were on a waiting list for SLH care at a Specialized Rehabilitation Center (CER) and had a prior diagnosis of phonological disorder, established in the center's care service, based on a comprehensive SLH assessment conducted prior to their inclusion in the research. This study applied the ABFW Phonology Test only to characterize the phonological profile of the sample and identify and describe the productive phonological processes at the time of data collection; it was not used as a diagnostic instrument.

CG was formed by convenience sampling, matched for sex and age range with SG.

All equipment was calibrated before data collection. The parents/guardians signed an informed consent form after receiving explanations about the study, and the children, after understanding it, signed an informed assent form.

The researchers surveyed their medical history, focusing on auditory and language aspects. The phonological assessment used the ABFW Test – Part A: Phonology, with 34 figures for naming and 39 words for imitation^(17)^. The samples were recorded with a smartphone (Samsung Galaxy A12, Android 11), phonetically transcribed, and analyzed based on the Percentage of Consonants Correct-Revised (PCC-R), classifying the severity of the disorder as mild, mild-moderate, moderate-severe, or severe^(18)^.

Although the severity of phonological disorder was characterized using PCC-R, the study used this data exclusively for clinically describing the sample and defining the groups. The analytical exploration of the relationship between phonological severity and the FFR electrophysiological parameters is part of another study being developed by the same research group and is, therefore, not the objective of this work. Thus, no correlational analyses were performed between PCC-R and FFR latency or amplitude in this manuscript.

The auditory canal inspection was performed using a Heine® Mini 3000 otoscope. Acoustic immittance measurements were obtained with the Interacoustics^®^ AT 235 analyzer, including type “A” tympanometry and the presence of ipsilateral and contralateral acoustic reflexes (according to Jerger et al.^(19)^).

Pure-tone and speech audiometry followed ANSI S3.6-2018 criteria, using the AD 629 audiometer (Interacoustics^®^), DD45 supra-aural headphones, and Vibrasom^®^ acoustic booth. The psychophysical method was used with 10-dB descending steps and 5-dB ascending steps, at 500, 1000, 2000, and 4000 Hz.

Auditory brainstem response (ABR) was applied with a click stimulus, duration of 100 µs, window of 10 ms, rate of 21.1 stimuli/second, EEG filter of 100–3000 Hz, and gain of 100,000. It also used 2000 stimuli in the rarefaction polarity, confirmed with the condensation polarity, at 80 dB HL. Electrodes were placed at Fz (positive), M1/M2 (reference), and FPz (ground), with impedance < 5 kΩ per electrode and < 2 kΩ between them. Results were considered abnormal in cases of increased latency (> 2 standard deviations) and/or absence of waves (CFFA, 2022)^(20)^.

FFR was performed using the speech stimulus /da/, provided by Dr. Nina Kraus's laboratory at Northwestern University. The examination was performed on the same equipment as the ABR, following the same electrode arrangement. Two scans of 3,000 stimuli at 80 dB HL were performed, presented at a rate of 10.9 stimuli/second, with a duration of 40 ms and an analysis window of 74.67 ms. During the acquisitions, the number of artifacts remained below 10%. A 100-Hz high-pass filter and a 2000-Hz low-pass filter were applied for wave filtering. All children included in the sample underwent FFR in both ears in monaural and binaural settings.

The children undergoing electrophysiological assessments were positioned in a comfortable reclining chair. During the examination, they remained awake (alert), watching a color, silent film and/or cartoon displayed via a mobile phone. The audiovisual content aimed to help maintain attention and relaxation without interfering with the auditory stimuli. Caregivers remained nearby, visible to the child, to provide greater emotional comfort and a sense of security throughout the procedure.

The FFR tracings were analyzed in the time domain, identifying the V, A, C, D, E, F, and O components and recording the absolute latencies and amplitudes. The analysis consisted of identifying the absolute latency, interpeak latency, and amplitude of each wave. At least two experienced researchers in the field analyzed all tracings to ensure the reliability of the results.

### Statistical analysis

Statistical analysis of data used the Statistical Package for the Social Sciences (SPSS), version 22. Descriptive statistics were initially performed for each dependent variable, calculating means, standard deviations, and standard error of the mean to characterize the study groups.

The homogeneity of variance test (Levene's Test) was applied to verify the assumption of homogeneity of variances between the groups. This test assesses whether the variances of the groups are significantly different, requiring a p-value > 0.05 to assume homogeneity and proceed with parametric tests.

A mixed factorial ANOVA was used to investigate the main effects of the group (phonological disorder vs. typical development), the stimulation condition (right monaural, left monaural, and binaural), and their interaction.

When statistically significant differences were identified in the ANOVA, a post-hoc analysis was performed using the Tukey HSD (honestly significant difference) test, which allows for the comparison of all conditions with each other and the identification of statistically relevant differences.

## RESULTS

This study included 42 children, 21 with typical development (CG) and 21 with phonological disorder (SG), matched for sex and age. The age range of the participants was 4 to 7 years and 11 months, with a mean age of 5 years and 9 months for the SG and 6 years for the CG; also, 57.14% were male, and 42.86% were female.

The most frequent phonological processes in SG were consonant cluster simplification (n = 20; 95.24%), final consonant simplification (n = 17; 80.95%), and liquid simplification (n = 16; 76.19%). As for severity, the sample ranged from mild to severe phonological disorders, with a higher occurrence of mild (n = 7; 33.33%) and moderate-severe (n = 7; 33.33%), as shown in Table 1.

**Table 1:** Characterization of the study group regarding sex, age, severity of phonological disorder, and phonological processes

Variables						Study Group		
						N		%
SEX		Males				12		57.14
		Females				9		42.86
			

AGE		4 years				9		42.86
		5 years				2		9.52
		6 years				6		28.57
		7 years				4		19.05
			
				
SEVERITY OF PHONOLOGICAL DISORDER		Mild				7		33.33
		Mild-Moderate				6		28.57
		Moderate-Severe				7		33.33
		Severe				1		4.77
			
				
PHONOLOGICAL PROCESSES		Consonant Cluster Simplification				20		95.24
		Liquid Simplification				16		76.19
		Final Consonant Simplification				17		80.95
		Velar Fronting				6		28.57
		Palatal Fronting				6		28.57
		Fricative Stopping				5		23.81
		Fricative Deafening				3		9.52
		Plosive Deafening				1		4.76
		Palatal Backing				5		23.81
		Velar Fricative Simplification				5		23.81
		Consonant Harmony				1		4.76
		Syllable Reduction				2		9.52
		Velar Backing				1		4.76
			

Source: Research data (2025)

The SG mean and standard deviation values for the analyzed variables are presented in Table 2, and those of CG are in Table 3. Levene's test was not significant, indicating that the variances between the groups and conditions are homogeneous, allowing the use of parametric tests.

**Table 2:** Descriptive measurements of FFR latencies for the right ear, left ear, and binaural sounds in the study and control groups

Variables	Latencies (n = 21)					
	Study Group			Control Group		
Right Ear	Mean	SD			Mean	SD
V	6.14	0.32			5.90	0.22
A	7.14	0.57			6.85	0.4
C	17.68	1.23			17.97	1.54
D	21.43	0.60			20.98	0.95
E	30.31	0.77			29.81	0.72
F	38.86	0.61			38.50	0.65
O	47.26	1.2			46.84	1.14
						
**Left Ear**	**Mean**	**SD**			**Mean**	**SD**
V	6.03	0.27			6.02	0.23
A	7.04	0.49			6.78	0.96
C	17.18	1.01			17.95	1.47
D	21.33	0.42			21.23	1.09
E	30.31	0.82			30.48	2.07
F	38.79	0.60			38.82	0.37
O	47.15	1.26			46.70	1.27
**Binaural**	**Mean**	**SD**			**Mean**	**SD**
V	6.09	0.26			5.95	0.20
A	7.07	0.48			6.94	0.38
C	17.60	1.39			17.95	1.37
D	21.14	0.67			21.32	0.24
E	30.12	0.75			30.01	0.52
F	38.77	0.79			38.72	0.32
O	47.10	1.14			47.00	0.99

Caption: SD = standard deviation

Source: Research data (2025)

**Table 3:** Descriptive measurements of FFR amplitudes for the right ear, left ear, and binaural sounds in the study and control groups

Variables	Amplitudes (n = 21)					
	Study Group			Control Group		
Right Ear	Mean	SD			Mean	SD
V	0.08	0.06			0.13	0.12
A	0.22	0.10			0.28	0.10
C	0.42	0.95			0.63	0.95
D	0.16	0.17			0.47	0.84
E	0.30	0.3			0.61	0.38
F	0.33	0.3			0.62	0.50
O	0.27	0.46			0.66	0.84
						
**Left Ear**	**Mean**	**SD**			**Mean**	**SD**
V	0.09	0.07			0.12	0.07
A	0.24	0.08			0.25	0.06
C	0.26	0.38			0.59	0.90
D	0.43	0.75			0.34	0.36
E	0.40	0.21			0.45	0.31
F	0.39	0.27			0.54	0.44
O	0.32	0.45			0.53	0.85
**Binaural**	**Mean**	**SD**			**Mean**	**SD**
V	0.21	0.10			0.24	0.10
A	0.30	0.15			0.34	0.10
C	1.33	1.77			2.98	3.53
D	0.52	1.23			0.17	0.31
E	0.82	0.79			1.39	1.19
F	0.75	0.83			1.73	2.48
O	1.04	2.13			2.4	3.72

Caption: SD = standard deviation

Source: Research data (2025)

The group with phonological disorders had higher latency values than the group with typically developing children in all stimulation conditions (right monaural, left monaural, and binaural). The mixed factorial ANOVA confirmed a significant effect of the group factor on latency (p = 0.006), indicating a statistically significant difference between the groups, with consistently higher wave V latencies in the three stimulation conditions among children with phonological disorders than among typically developing ones.

The difference between the three stimulation conditions was minimal, which explains the absence of a significant effect of the condition on latency (p = 0.991), indicating that the form of stimulus presentation (monaural or binaural) did not significantly influence the response. Furthermore, the interaction between group and condition was also not significant (p = 0.131), suggesting that the groups did not respond differently to the different forms of stimulation.

Although not statistically significant, the latency of the C wave was lower in the group with phonological disorder than in the group with typical development. The C wave represents the transition between the consonant and the vowel in the speech stimulus.

Moreover, the group with phonological disorder had smaller amplitudes than the group with typical development in all stimulation conditions, although this difference was not statistically significant. The exception was for the amplitude of the D wave in the binaural condition, which was lower in the group with typical development, also without a statistical difference. Consequently, the analysis revealed that the group effect was not significant (p = 0.055), indicating that the FFR amplitude was not sensitive to differentiate the groups in any of the conditions tested.

Multiple comparisons, performed using Tukey's post-hoc test, showed that the binaural condition presented significantly larger amplitudes than the monaural conditions (p < 0.001), suggesting a binaural summation effect. The only exception was the amplitude of the D wave in the binaural condition of the typically developing group, which did not follow this trend. Furthermore, no significant differences were observed between the right and left monaural conditions (p > 0.05).

## DISCUSSION

This study aimed to analyze whether FFR electrophysiological measurements in the time domain differed between children with phonological disorder and those with typical development, considering monaural and binaural stimulation conditions. The analysis focused on the latency and amplitude measurements of the FFR components, seeking to understand whether neural coding of speech is altered in children with phonological disorder.

The study results demonstrated longer latencies in children with phonological disorder than in children with typical development in all stimulation conditions, with a statistically significant effect of the "group" factor on the latency of wave V. This finding aligns with the hypothesis of impairment in the temporal coding of auditory stimuli at the subcortical level. These responses are similar to the results of a study^(2)^ that identified an increase in the latencies of waves V, A, C, F, and O and a greater slope of the V-A complex in children with phonological disorder, which signals a possible difficulty in the neural coding of transient speech segments, possibly compromising sound detection and discrimination.

Furthermore, the findings of another study^(8)^ were similar to those of this research, as they showed significantly longer latencies of waves V and A in children with phonological disorder and amplitudes without statistical differences. This suggests that children with phonological disorder may process auditory information differently in the initial phases, compared to typically developing children ​​which could be attributed to a delay in the time the auditory system takes to process sound stimuli, indicating less efficiency in neuronal synchronization of a complex sound, such as speech.

The agreement between the findings suggests a consistent trend of delay in FFR responses in children with phonological disorder, mainly in the initial components of the stimulus, implying the transient characteristics of the sound. Thus, the increase in latencies can be interpreted as an objective marker of immaturity or alteration in temporal coding at the subcortical level in this population^(2,8)^. However, these findings should not be interpreted in isolation, since the FFR does not replace clinical, phonological, and behavioral assessment; rather, it should be understood as a complementary examination in the diagnostic context.

Impaired speech sound encoding interferes with the development of linguistic skills, since the learning of phonological, syntactic, and semantic elements depends directly on the integrity of the neural mechanisms responsible for auditory encoding. In this context, the increase in latencies in FFR responses observed in this study reinforces the hypothesis that deficits in temporal encoding compromise auditory processing at cortical and subcortical levels, negatively affecting the ability of the auditory system to discriminate and perceive speech sounds. These alterations can impact the development of oral language, hindering the acquisition and refinement of linguistic skills^(21)^.

In this context, the findings reinforce that FFR latency can assist in the differential diagnosis of phonological disorders, as well as in therapeutic monitoring, making it possible to track possible changes in the neural encoding of speech in SLH interventions.

The lower C-wave latency observed in the group with phonological disorder, although without a statistically significant difference, may be related to the characteristics of a basic phonological alteration, in which children with phonological disorder present systematic consonant substitutions. Vowels, in turn, being phonemes of lower articulatory complexity, tend to present adequate productions^(22)^. Thus, the lower C-wave latency values ​​may suggest that auditory processing in the transition between consonant and vowel is relatively preserved.

Regarding amplitude, although the group with phonological disorder generally presented lower values than the group with typical development, this difference was not statistically significant. This finding is consistent with a study^(8)^ that also found no statistically relevant differences between the groups. The absence of a group effect on amplitude suggests that this parameter may not be sensitive enough to distinguish children with and without phonological disorder.

According to a systematic review^(23)^ that analyzed FFR responses in children with and without oral language disorders, amplitude measures were more variable across studies, with results ranging from reduced amplitudes to the absence of significant differences between groups. In contrast, latency parameters were more consistent, with a trend toward increased latencies in most studies with populations that presented oral language disorders.

Thus, as study findings^(21,24)^ point out, the most frequent FFR alterations in populations with language disorders are generally associated with temporal encoding (evidenced by increased latencies) and not necessarily with the intensity of the neural response, observed in amplitudes. These findings suggest that, while FFR latency may be an electrophysiological marker for phonological disorders, amplitude, in turn, may not be the most discriminative parameter to alert about possible language and speech disorders.

From a clinical point of view, these findings reinforce that FFR should be used as an objective and complementary tool, providing additional information on the efficiency of neural speech coding, without replacing clinical and behavioral assessments.

Moreover, when relating amplitude to the form of stimulus presentation, children had greater amplitude responses in the binaural condition, suggesting a binaural summation effect. This finding indicates that simultaneous stimulation in both ears may favor the identification of the neural response, possibly due to the synchronous integration of auditory signals along the auditory pathway^(10,24)^.

The effect of the stimulation condition was highly significant, indicating that binaural presentation influences the amplitude of the responses. Furthermore, the interaction between group and condition was not significant, which demonstrates that binaural summation exerts a positive effect on the amplification of the neural response, regardless of the group. The absence of differences between sides in the monaural condition suggests that unilateral stimulation generates similar responses, regardless of the ear stimulated. These findings may justify the option adopted in some studies with auditory evoked potentials of presenting the stimulus in monaural conditions, either alternately in both ears or exclusively in the right ear^(2,7-9,25)^.

The preference for the right ear in monaural presentations is based on left hemispheric dominance for language processing. Considering that auditory information predominantly travels through crossed pathways in the central nervous system, linguistic stimuli presented in the right ear are directed to the left hemisphere, favoring a faster and more efficient neural encoding of speech sounds^(11,26)^.

The absence of a significant effect of the stimulation condition (monaural or binaural) on latency suggests that the form of stimulus presentation does not substantially interfere with the processing of the temporal aspects of speech. This indicates that the integrity of neural responses in children with phonological disorder may remain altered, regardless of the ear tested or simultaneous stimulation. As pointed out^(11)^, although FFR is an auditory evoked potential sensitive to sensory input (i.e., to how sound reaches the auditory system), stimulus presentation in both ears may optimize the robustness of the neural response but is not sufficient to compensate for specific deficits in auditory processing and encoding in children with phonological disorder.

### Implications for research and limitations of the study

The study results indicate that FFR can be an objective marker in the investigation of changes in auditory coding in children with phonological disorders, especially in the transient characteristics of sound, evidenced by the increased latencies of the components. However, further research can deepen the analysis of FFR, both in the time and frequency domains, in children with phonological disorders and other SSDs to compare the characteristics of auditory responses in the different types of these disorders. This can provide a better understanding of the similarities and discrepancies in auditory processing in children with SSDs, thus helping to improve therapeutic intervention strategies in these disorders.

Longitudinal studies should be carried out to monitor the maturation of the auditory pathway and especially to evaluate the advances in the coding of auditory information with SLH interventions. This would enable the investigation into whether progress in the linguistic and auditory skills of children with phonological disorders is associated with the normalization of FFR responses, which could serve as an objective marker of therapeutic efficacy and help define the therapeutic goals to be stimulated.

Future studies can also correlate the severity of the phonological disorder and the number of phonological processes with latency and amplitude findings, investigating whether children with greater phonological impairment and greater severity have more altered FFR responses and associating FFR electrophysiological findings with behavioral data obtained through tests that assess central auditory processing.

It is important to consider that socioeconomic and environmental factors, such as maternal education level, family income, and school type, exert a significant influence on children's auditory and linguistic development. Studies indicate that environments with less linguistic stimulation and greater social vulnerability can negatively impact the development of auditory and language skills, regardless of specific neurophysiological alterations^(27)^.

Not controlling or matching these variables between the groups represents a limitation of the present study and should be considered in the interpretation of the results, since such factors may act as intervening variables in children’s auditory and linguistic performance.

## CONCLUSION

The study results indicate that children with phonological disorder exhibit impairment in the neural encoding of speech sounds, evidenced by increased latencies (significantly longer in wave V of the FFR) than typically developing children, regardless of the stimulation condition. Additionally, binaural stimulation promoted shorter latencies and larger amplitudes than monaural conditions, suggesting a binaural summation effect.
