# Supplementary material for: Analysis of neural coding of speech in monaural and binaural conditions in children with speech sound disorder
Source: Codas. 2026 Jul 10;38(5):e20250421. doi: 10.1590/2317-1782/e20250421en (PMC13440571; doi:10.1590/2317-1782/e20250421en)
Supplement: Translation

# Translation: Análise da codificação neural de fala em condições mono e binaural em crianças com transtorno dos sons da fala

**DOI:** 10.1590/2317-1782/e20250421pt

**Authors:** Marciana da Costa Carlos, Thaís Nobre Uchôa Souza, Ranilde Cristiane Cavalcante Costa, Aline Tenório Lins Carnaúba

**Affiliations:** 1 Programa Associado de Pós-graduação em Fonoaudiologia, Universidade Estadual de Ciências da Saúde de Alagoas – UNCISAL - Maceió (AL), Brasil.; 2 Departamento de Fonoaudiologia, Universidade Estadual de Ciências da Saúde de Alagoas – UNCISAL - Maceió (AL), Brasil.

**Keywords:** Potencial Evocado Auditivo, Transtorno Fonológico, Crianças, Fala, Eletrofisiologia

## Abstract

**Objetivo:**

Analisar se crianças com transtorno fonológico apresentam alterações na codificação neural da fala, por meio do Frequency Following Response (FFR), nas condições monoaural e binaural.

**Método:**

Estudo analítico, observacional e transversal, com amostra de 21 crianças com Transtorno Fonológico e 21 com desenvolvimento típico. Os indivíduos foram submetidos aos seguintes procedimentos: anamnese, avaliação fonológica, inspeção do meato acústico externo, avaliação audiológica e eletrofisiológica, realizados em uma ou duas sessões. O FFR foi realizado com estímulo de fala /da/, em duas varreduras de 3000 estímulos de 80 dB NA, com taxa de 10,9 estímulos/segundo, duração de 40 ms e janela de análise de 74,677 ms.

**Resultados:**

As crianças com Transtorno Fonológico apresentaram maiores valores de latência em todas condições em comparação com o Desenvolvimento Típico, com significância estatística na latência da onda V e amplitudes menores em relação às com desenvolvimento típico, porém sem significância estatística. Na forma de apresentação do estímulo, a condição binaural apresentou amplitudes significativamente maiores em relação às monoaurais, sugerindo a presença de um efeito de somação binaural**.**

**Conclusão:**

As crianças com transtorno fonológico apresentam alterações na codificação da fala, evidenciadas pelo atraso nas respostas do FFR nas condições monoaural e binaural.

## INTRODUÇÃO

A integridade das vias sensoriais é essencial para o desenvolvimento infantil, uma vez que o processamento das informações auditivas permite a discriminação de pistas acústicas importantes para a aquisição fonológica e o aprimoramento das habilidades auditivas. Dessa forma, tanto a via auditiva periférica quanto a central são fundamentais para a aquisição da linguagem oral^(1)^.

Nesse contexto, a maturação do Sistema Nervoso Auditivo Central (SNAC) desempenha papel crucial para a qualidade do processamento auditivo e quando prejudicada, pode resultar em déficits perceptivos e transtornos de comunicação^(1)^.

O desenvolvimento da linguagem, especialmente das habilidades fonológicas das crianças, depende da percepção precisa dos sons da fala. O processamento refinado das características acústicas, particularmente das rápidas mudanças temporais na dinâmica da fala, é essencial para esse desenvolvimento^(2)^. Diante disso, quando há uma desorganização em algum desses processos relacionados à produção e percepção dos sons, ocorre uma dificuldade na apropriação do sistema fonológico, o que pode acarretar a omissão ou substituição de fonemas^(3)^, como observado nos Transtornos dos Sons da Fala (TSF).

TSF constitui um termo abrangente que se refere a quaisquer dificuldades ligadas à articulação ou à representação fonológica dos sons da fala, resultando em uma diminuição da inteligibilidade. Dentre os subtipos de TSF, o transtorno fonológico relaciona-se a alterações na consolidação do sistema fonológico, caracterizadas por substituições e/ou omissões recorrentes de fonemas, que geralmente afetam uma classe de sons e persistem em faixas etárias em que isso já não seria esperado^(4)^.

Estudos recentes evidenciaram que crianças com transtorno fonológico apresentaram resultados aquém do esperado para idade nos testes comportamentais de processamento auditivo central^(5,6)^ e testes eletrofisiológicos^(7-9)^. Dentre os exames eletrofisiológicos, o *Frequency Following Response* (FFR) é um teste que avalia a codificação da fala por meio da atividade sincronizada dos neurônios nas vias auditivas, com foco no tronco encefálico, uma área vital para a linguagem e a audição. Estudos recentes mostram que o FFR pode ser uma ferramenta valiosa para o diagnóstico diferencial de transtornos relacionados ao processamento auditivo, linguagem e fala^(8)^.

Devido a capacidade do sistema auditivo humano de processar as diferenças dos sinais acústicos que chegam a uma ou a ambas as orelhas, o FFR pode ser realizado nas condições de estimulação monoaural (direita e esquerda) e binaural (simultaneamente nas duas orelhas). A estimulação monoaural investiga a codificação do estímulo de fala em apenas uma orelha – possibilitando a análise de assimetrias auditivas ou lateralizações. Já a condição binaural refere-se a apresentação dos estímulos simultaneamente nas duas orelhas, possibilitando uma percepção mais intensa do som. A análise comparativa entre essas duas condições é importante na avaliação da eficiência da codificação auditiva isolada, assim como da integração neural entre os hemisférios cerebrais, expandindo a compreensão dos mecanismos presentes no processamento da fala e linguagem ^(10,11)^.

Além disso, dentre as medidas extraídas dos componentes analisados no FFR, encontram-se as latências e amplitudes, que geram dados relevantes sobre o funcionamento do sistema auditivo. Com isso, a latência está relacionada ao tempo da resposta neural diante de um estímulo auditivo, o que permite analisar a sincronização temporal durante a codificação neural de um som complexo. Em contrapartida, a amplitude indica a robustez e a força da resposta elétrica gerada frente a estímulo auditivo, recebendo influência da sincronização neural. As alterações nessas medidas podem indicar disfunções na codificação temporal e integridade das vias auditivas. Assim, a análise das latências e amplitudes do FFR pode contribuir para a análise das bases neurobiológicas dos transtornos de fala e linguagem, trazendo implicações relevantes para diagnóstico precoce, como também para o início das intervenções fonoaudiológicas^(12)^.

Diferentemente dos testes comportamentais^(13)^, que dependem da resposta e da concentração da criança, o FFR oferece uma medida objetiva e precisa de como o cérebro processa sons auditivos complexos, como a fala^(14)^. Ele permite identificar possíveis déficits na percepção auditiva, frequentemente presentes em crianças com transtorno fonológico, uma vez que esse transtorno envolve dificuldades na percepção e discriminação dos traços distintivos — responsáveis por transmitir propriedades acústicas dos sons da fala, como frequência, sonoridade e padrões temporais. Portanto, os achados do FFR podem contribuir para o entendimento da codificação auditiva no transtorno fonológico e consequentemente auxiliar nas estratégias terapêuticas para redução e eliminação desse transtorno^(2)^.

Contudo, apesar da crescente utilização do FFR na investigação dos transtornos de linguagem e fala^(15)^, ainda são poucos os estudos que exploram as diferenças entre as condições de estimulação monoaural e binaural nessas populações, inclusive em crianças com transtorno fonológico. Assim, considerando que o FFR permite uma análise detalhada e objetiva da codificação neural dos sons da fala e que crianças com transtorno fonológico apresentam comprometimento na percepção dos contrastes da língua, o objetivo do presente estudo é analisar se crianças com transtorno fonológico apresentam alterações na codificação neural da fala, por meio do FFR, nas condições monoaural e binaural.

## MÉTODOS

Trata-se de um estudo analítico, observacional e transversal, aprovado pelo Comitê de Ética em Pesquisa (CEP) da Universidade Estadual de Ciências da Saúde de Alagoas (UNCISAL) com número do parecer N. 6.692.677 e desenvolvido no Laboratório de Audição e Tecnologia da UNCISAL. Todas as normas e diretrizes para pesquisa com seres humanos foram cumpridas, de acordo com a Resolução 466/12 do Conselho Nacional de Saúde do Brasil.

A amostra foi constituída por dois grupos: Grupo Estudo – GE, formado por 21 crianças com transtorno fonológico e Grupo Controle – GC, composto por 21 crianças com desenvolvimento típico, pareados conforme sexo e idade; definida por meio da comparação entre médias resultando em 21 crianças para cada grupo. Os parâmetros utilizados para o cálculo do tamanho da amostra foram: Alfa = 0,05; Beta= 0,1; Desvio padrão = 0,5; Diferença entre os grupos = 0,8, conforme os resultados encontrados por Gonçalves^(16)^.

Ambos os grupos (GE e GC) apresentaram os seguintes critérios de inclusão: limiares auditivos tonais dentro do padrão de normalidade (até 15 dB NA em oitavas de 500 a 4.000 Hz); inspeção visual normal do canal auditivo externo; presença de timpanogramas tipo “A” e latências absolutas e interpicos referentes às ondas I, III e V dentro dos critérios de normalidade bilateralmente. Os critérios de exclusão de ambos os grupos foram: mais de três infecções de ouvido no ano corrente; diagnóstico de Desordem do Espectro da Neuropatia Auditiva (DENA); síndromes genéticas, disfunção neurológica, psíquica ou deficiência intelectual, e alterações estruturais nos órgãos fonoarticulatórios que causem alterações na fala (tais como fissura labiopalatina ou frênulo lingual curto).

Referente aos critérios específicos do GE, as crianças apresentavam processos fonológicos produtivos na fala. As crianças incluídas no estudo encontravam-se em lista de espera para atendimento fonoaudiológico em um Centro Especializado em Reabilitação (CER) e possuíam diagnóstico prévio de Transtorno Fonológico, estabelecido no contexto assistencial do serviço, a partir de avaliação fonoaudiológica abrangente realizada previamente à inclusão na pesquisa. No âmbito do presente estudo, a Prova de Fonologia do ABFW foi aplicada apenas para caracterização do perfil fonológico da amostra, com o objetivo de identificar e descrever os processos fonológicos produtivos no momento da coleta de dados, não sendo utilizada como instrumento diagnóstico.

O GC foi constituído por amostragem por conveniência, pareadas em sexo e faixa etária com o GE.

Antes da coleta, todos os equipamentos foram calibrados. Os responsáveis assinaram o Termo de Consentimento Livre e Esclarecido (TCLE) após receberem as explicações sobre o estudo, e as crianças, após entendimento, assinaram o Termo de Assentimento Livre e Esclarecido (TALE).

Foi realizada uma anamnese com foco em aspectos auditivos e de linguagem. A avaliação fonológica utilizou o Teste ABFW – Parte A: Fonologia, com 34 figuras para nomeação e 39 vocábulos para imitação^(17)^. As amostras foram gravadas com *smartphone* (Samsung Galaxy A12, Android 11), transcritas foneticamente e analisadas com base no índice Porcentagem de Consoantes Corretas-Revisado (PCC-R), classificando a gravidade do transtorno em leve, leve-moderado, moderado-grave ou grave^(18)^.

Embora a gravidade do Transtorno Fonológico tenha sido caracterizada por meio do índice PCC-R, esse dado foi utilizado neste estudo exclusivamente para a descrição clínica da amostra e definição dos grupos. A exploração analítica da relação entre gravidade fonológica e os parâmetros eletrofisiológicos do FFR integra um outro estudo em desenvolvimento pelo mesmo grupo de pesquisa, não sendo, portanto, objetivo do presente trabalho. Assim, não foram realizadas análises correlacionais entre PCC-R e latência ou amplitude do FFR neste manuscrito.

A inspeção do conduto auditivo foi feita com otoscópio Heine® Mini 3000. As medidas de imitância acústica foram obtidas com o analisador AT 235 da Interacoustics®, incluindo curva timpanométrica tipo “A” e presença de reflexos acústicos ipsi e contralaterais (conforme Jerger et al.^(19)^).

Na audiometria tonal e vocal, realizada com o audiômetro AD 629 (Interacoustics®), fones supra-aurais DD45 e cabine acústica Vibrasom®, seguiram-se os critérios ANSI S3.6-2018. Utilizou-se o método psicofísico com passos descendentes de 10 dB e ascendentes de 5 dB, nas frequências de 500, 1000, 2000 e 4000 Hz.

Os Potenciais Evocados Auditivos de Tronco Encefálico (PEATE) foram aplicados com estímulo clique, duração de 100 µs, janela de 10 ms, velocidade de 21,1 estímulos/segundo, filtro EEG de 100–3000 Hz e ganho de 100.000. Foram usados 2000 estímulos na polaridade rarefeita, confirmados com a condensada, a 80 dB NA. Eletrodos foram colocados em Fz (positivo), M1/M2 (referência) e FPz (terra), com impedância <5 kΩ por eletrodo e <2 kΩ entre eles. Resultados foram considerados alterados em casos de latência aumentada (>2 desvios padrão) e/ou ausência de ondas (CFFA, 2022)^(20)^.

O FFR foi realizado com o estímulo de fala /da/, disponibilizado pelo laboratório da Dra. Nina Kraus da Northwestern University. O exame foi realizado no mesmo equipamento do PEATE, seguindo a mesma organização dos eletrodos. Foram realizadas duas varreduras de 3.000 estímulos a 80 dB NA, apresentados a uma taxa de 10,9 estímulos/segundo, com duração de 40 ms e uma janela de análise de 74,67 ms. Durante as aquisições, o número de artefatos manteve-se abaixo de 10%. Para filtragem das ondas, aplicou-se filtro passa-alto de 100 Hz e passa-baixo de 2000 Hz. Todas as crianças incluídas na amostra realizaram o FFR em ambas as orelhas de forma monoaural e binaural.

As crianças submetidas à avaliação eletrofisiológica foram posicionadas em uma poltrona reclinável e confortável. Durante o exame, permaneceram acordadas - em estado de vigília - e assistindo a um filme e/ou desenho, colorido e sem áudio, exibido por meio de um aparelho celular. O conteúdo audiovisual teve como objetivo auxiliar na manutenção da atenção e do relaxamento, sem interferir nos estímulos auditivos apresentados. Os responsáveis permaneceram próximos, em um local visível à criança, a fim de proporcionar maior conforto emocional e sensação de segurança ao longo do procedimento.

Os traçados do FFR foram analisados no domínio do tempo, com identificação dos componentes V, A, C, D, E, F, O, com registro das latências absolutas e das amplitudes. A análise consistiu na identificação da latência absoluta, interpico e amplitude de cada onda. Todos os traçados foram analisados por, pelo menos, dois pesquisadores experientes na área, a fim de garantir a confiabilidade dos resultados.

### Análise estatística

Para a análise estatística dos dados, foi utilizado o software *Statistical Package for the Social Sciences* (SPSS), versão 22. Inicialmente, foi realizada a estatística descritiva para cada variável dependente, com cálculo de médias, desvios padrão e erro padrão da média, a fim de caracterizar os grupos de estudo.

Para verificar a suposição de homogeneidade das variâncias entre os grupos, foi aplicado o Teste de Homogeneidade de Variância (Teste de Levene). Esse teste avalia se as variâncias dos grupos são significativamente diferentes, sendo necessário um valor de p > 0,05 para assumir homogeneidade e prosseguir com testes paramétricos.

Para investigar os efeitos principais do grupo (Transtorno Fonológico vs. Desenvolvimento Típico) e da condição de estimulação (monoaural direita, monoaural esquerda e binaural), bem como sua interação, foi utilizada uma ANOVA fatorial mista.

Quando identificadas diferenças estatisticamente significativas na ANOVA, foi realizada uma análise post-hoc com o teste Tukey HSD (Honestly Significant Difference), que permite comparar todas as condições entre si e identificar diferenças estatisticamente relevantes.

## RESULTADOS

O presente estudo incluiu 42 crianças, sendo 21 com desenvolvimento típico (GC) e 21 com transtorno fonológico (GE), pareadas em sexo e idade. A faixa etária dos participantes foi de 4 a 7 anos e 11 meses, com média de idade de 5 anos e 9 meses para o GE e 6 anos para o GC, sendo 57,14% do sexo masculino e 42,86% do feminino.

Para o grupo com transtorno fonológico, os processos fonológicos mais frequentes foram a simplificação de encontro consonantal (n=20; 95,24%), simplificação de consoante final (n=17; 80,95%) e a simplificação de líquida (n=16; 76,19%). Quanto a gravidade do transtorno fonológico, a amostra variou de leve a grave, com maior ocorrência do TF leve (n=7; 33,33%) e moderado-grave (n=7; 33,33%), conforme a Tabela 1.

**Tabela 1:** Caracterização do grupo de estudo quanto às variáveis gênero, idade, gravidade do transtorno fonológico e processos fonológicos

**Variáveis**						**Grupo de Estudo**		
						**N**		%
GÊNERO		Masculino				12		57,14
		Feminino				9		42,86
			

IDADE		4 anos				9		42,86
		5 anos				2		9,52
		6 anos				6		28,57
		7 anos				4		19,05
			
				
GRAVIDADE DO TRANSTORNO FONOLÓGICO		Leve				7		33,33
		Levemente-Moderado				6		28,57
		Moderado-Grave				7		33,33
		Grave				1		4,77
			
				
PROCESSOS FONOLÓGICOS		Simplificação de Encontro Consonantal				20		95,24
		Simplificação de Líquida				16		76,19
		Simplificação de Consoante Final				17		80,95
		Frontalização de Velar				6		28,57
		Frontalização de Palatal				6		28,57
		Plosivação de Fricativa				5		23,81
		Ensurdecimento de Fricativa				3		9,52
		Ensurdecimento de Plosiva				1		4,76
		Posteriorização para Palatal				5		23,81
		Simplificação de Fricativa Velar				5		23,81
		Harmonia Consonantal				1		4,76
		Redução de Sílaba				2		9,52
		Posteriorização para Velar				1		4,76
			

Fonte: Dados da pesquisa (2025)

Os valores de média e desvios padrão do GE para as variáveis analisadas são apresentados na Tabela 2 e do GC na Tabela 3. O teste de Levene não foi significativo, indicando que as variâncias entre os grupos e condições são homogêneas, permitindo o uso de testes paramétricos.

**Tabela 2:** Medidas descritivas das latências do FFR para a orelha direita, esquerda e binaural dos grupos estudo e controle

Variáveis	Latências (n = 21)					
	Grupo Estudo			Grupo Controle		
Orelha Direita	Média	DP			Média	DP
V	6,14	0,32			5,90	0,22
A	7,14	0,57			6,85	0,4
C	17,68	1,23			17,97	1,54
D	21,43	0,60			20,98	0,95
E	30,31	0,77			29,81	0,72
F	38,86	0,61			38,50	0,65
O	47,26	1,2			46,84	1,14
						
**Orelha Esquerda**	**Média**	**DP**			**Média**	**DP**
V	6,03	0,27			6,02	0,23
A	7,04	0,49			6,78	0,96
C	17,18	1,01			17,95	1,47
D	21,33	0,42			21,23	1,09
E	30,31	0,82			30,48	2,07
F	38,79	0,60			38,82	0,37
O	47,15	1,26			46,70	1,27
**Binaural**	**Média**	**DP**			**Média**	**DP**
V	6,09	0,26			5,95	0,20
A	7,07	0,48			6,94	0,38
C	17,60	1,39			17,95	1,37
D	21,14	0,67			21,32	0,24
E	30,12	0,75			30,01	0,52
F	38,77	0,79			38,72	0,32
O	47,10	1,14			47,00	0,99

Legenda: DP = desvio padrão

Fonte: Dados da pesquisa (2025)

**Tabela 3:** Medidas descritivas das amplitudes do FFR para a orelha direita, esquerda e binaural dos grupos estudo e controle

Variáveis	Amplitudes (n = 21)					
	Grupo Estudo			Grupo Controle		
Orelha Direita	Média	DP			Média	DP
V	0,08	0,06			0,13	0,12
A	0,22	0,10			0,28	0,10
C	0,42	0,95			0,63	0,95
D	0,16	0,17			0,47	0,84
E	0,30	0,3			0,61	0,38
F	0,33	0,3			0,62	0,50
O	0,27	0,46			0,66	0,84
						
**Orelha Esquerda**	**Média**	**DP**			**Média**	**DP**
V	0,09	0,07			0,12	0,07
A	0,24	0,08			0,25	0,06
C	0,26	0,38			0,59	0,90
D	0,43	0,75			0,34	0,36
E	0,40	0,21			0,45	0,31
F	0,39	0,27			0,54	0,44
O	0,32	0,45			0,53	0,85
**Binaural**	**Média**	**DP**			**Média**	**DP**
V	0,21	0,10			0,24	0,10
A	0,30	0,15			0,34	0,10
C	1,33	1,77			2,98	3,53
D	0,52	1,23			0,17	0,31
E	0,82	0,79			1,39	1,19
F	0,75	0,83			1,73	2,48
O	1,04	2,13			2,4	3,72

Legenda: DP = desvio padrão

Fonte: Dados da pesquisa (2025)

Observou-se que o grupo com transtorno fonológico apresentou maiores valores de latência em comparação ao grupo com desenvolvimento típico em todas as condições de estimulação (monoaural direita, monoaural esquerda e binaural). A ANOVA fatorial mista confirmou um efeito significativo do fator grupo sobre a variável latência (p = 0,006), indicando uma diferença estatisticamente significativa entre os grupos, com latências da onda V consistentemente maiores nas três condições de estimulação para as crianças com transtorno fonológico em relação às crianças com desenvolvimento típico.

A diferença entre as três condições de estimulação foi mínima, o que explica a ausência de efeito significativo da condição sobre a latência (p = 0,991), indicando que a forma de apresentação do estímulo (monoaural ou binaural) não influenciou significativamente a resposta. Além disso, a interação entre grupo e condição também não foi significativa (p = 0,131), sugerindo que os grupos não responderam de maneira diferente às diferentes formas de estimulação.

Embora sem diferença estatisticamente significativa, a latência da onda C foi menor no grupo com transtorno fonológico em comparação ao grupo com desenvolvimento típico. A onda C é um componente que representa a transição entre a consoante e a vogal no estímulo de fala.

Em relação à variável amplitude, observou-se que o grupo com TF apresentou amplitudes menores em comparação ao grupo com desenvolvimento típico em todas as condições de estimulação, embora essa diferença não tenha sido estatisticamente significativa. A exceção foi para a amplitude da onda D na condição binaural, que foi inferior no grupo com desenvolvimento típico, também sem diferença estatística. Como consequência, a análise revelou que o efeito do grupo não foi significativo (p = 0,055), indicando que a amplitude do FFR não foi sensível para diferenciar os grupos em nenhuma das condições testadas.

As comparações múltiplas, realizadas pelo teste post-hoc de Tukey, mostraram que a condição binaural apresentou amplitudes significativamente maiores em relação às condições monoaurais (p < 0,001), sugerindo a presença de um efeito de somação binaural. A única exceção foi a amplitude da onda D na condição binaural do grupo com desenvolvimento típico, que não seguiu essa tendência. Além disso, não foram observadas diferenças significativas entre as condições monoaurais direita e esquerda (p > 0,05).

## DISCUSSÃO

Este estudo teve como objetivo analisar se há diferenças nas medidas eletrofisiológicas do FFR no domínio do tempo em crianças com transtorno fonológico e com desenvolvimento típico, considerando as condições de estimulação monoaural e binaural. A análise concentrou-se nas medidas de latência e amplitude dos componentes do FFR, buscando compreender se há alterações na codificação neural da fala em crianças com transtorno fonológico.

Os resultados obtidos no presente estudo demonstraram latências maiores nas crianças com transtorno fonológico em relação às crianças com desenvolvimento típico em todas as condições de estimulação, com efeito estatisticamente significativo do fator “grupo” na latência da onda V. Esse achado alinha-se à hipótese de um prejuízo na codificação temporal dos estímulos auditivos a nível subcortical. Essas respostas são semelhantes aos resultados da pesquisa^(2)^, que identificaram um aumento nas latências das ondas V, A, C, F e O e maior inclinação do complexo V-A nas crianças com transtorno fonológico, o que sinaliza uma possível dificuldade na codificação neural dos segmentos transitórios da fala, o que pode comprometer a detecção e discriminação dos sons.

Além disso, os achados do estudo^(8)^ foram semelhantes aos desse estudo, visto que evidenciaram latências significativamente maiores das ondas V e A em crianças com transtorno fonológico e amplitudes sem diferenças estatísticas, o que sugere que crianças com transtorno fonológico podem processar as informações auditivas de forma diferente nas fases iniciais, em comparação às crianças com desenvolvimento típico – o que poderia ser atribuído a um atraso no tempo que o estímulo sonoro leva para ser processado pelo sistema auditivo, indicando uma menor eficiência na sincronização neuronal diante de um som complexo, como a fala.

A concordância entre os achados sugere uma tendência consistente de atraso nas respostas do FFR em crianças com transtorno fonológico, principalmente nos componentes iniciais do estímulo, implicando nas características transitórias do som. Dessa forma, o aumento das latências pode ser interpretado como um marcador objetivo de imaturidade ou alteração na codificação temporal a nível subcortical nessa população^(2,8)^. No entanto, é importante destacar que tais achados não devem ser interpretados de forma isolada, uma vez que o FFR não substitui a avaliação clínica, fonológica e comportamental, mas deve ser compreendido como um exame complementar no contexto diagnóstico.

O comprometimento na codificação dos sons da fala interfere no desenvolvimento das habilidades linguísticas, visto que o aprendizado de elementos fonológicos, sintáticos e semânticos dependem diretamente da integridade dos mecanismos neurais responsáveis pela codificação auditiva. Nesse contexto, o aumento das latências nas respostas do FFR observado neste estudo reforça a hipótese de que déficits na codificação temporal comprometem o processamento auditivo em níveis corticais e subcorticais, afetando negativamente a capacidade do sistema auditivo de discriminar e perceber os sons da fala. Essas alterações podem impactar o desenvolvimento da linguagem oral, dificultando a aquisição e o refinamento das habilidades linguísticas^(21)^.

Nesse contexto, os achados reforçam que a latência do FFR pode auxiliar no diagnóstico diferencial de transtornos fonológicos, bem como no monitoramento terapêutico, permitindo acompanhar possíveis modificações na codificação neural da fala ao longo das intervenções fonoaudiológicas.

A menor latência da onda C observada no grupo com transtorno fonológico, embora sem diferença estatisticamente significativa, pode estar relacionada às características de uma alteração de base fonológica, em que crianças com transtorno fonológico apresentam trocas sistemáticas de consoantes. As vogais, por sua vez, por serem fonemas de menor complexidade articulatória, tendem a apresentar produções adequadas^(22)^. Assim, os menores valores de latência da onda C podem sugerir que o processamento auditivo na transição entre consoante e vogal está relativamente preservado.

No que se refere à variável amplitude, observou-se que, embora o grupo com transtorno fonológico tenha apresentado valores geralmente menores em comparação ao grupo com desenvolvimento típico, essa diferença não foi estatisticamente significativa. Esse achado é consistente com o estudo^(8)^, que também não encontrou diferenças estatisticamente relevantes entre os grupos. A ausência de efeito do grupo na amplitude sugere que esse parâmetro pode não se configurar sensível para distinguir crianças com e sem transtorno fonológico.

Conforme a revisão sistemática^(23)^, que analisou as respostas do FFR em crianças com e sem transtornos de linguagem oral, as medidas de amplitude mostraram-se mais variáveis entre os estudos, com resultados que oscilaram entre a redução das amplitudes e a ausência de diferenças significativas entre os grupos. Em contrapartida, os parâmetros de latência apresentaram maior consistência, sendo observada uma tendência ao aumento das latências na maioria dos estudos com populações que apresentavam transtornos de linguagem oral.

Com isso, conforme apontam os achados de ^(21,24)^, as alterações mais frequentes no FFR em populações com transtornos de linguagem estão geralmente associadas à codificação temporal — evidenciada pelo aumento das latências — e não necessariamente à intensidade da resposta neural, observada nas amplitudes. Esses achados sugerem que, enquanto a latência do FFR pode ser um marcador eletrofisiológico para transtornos fonológicos, a amplitude, por sua vez, pode não se configurar como o parâmetro mais discriminativo para alertar sobre possíveis transtornos de linguagem e fala.

Do ponto de vista clínico, esses achados reforçam que o FFR deve ser utilizado como uma ferramenta objetiva e complementar, fornecendo informações adicionais sobre a eficiência da codificação neural da fala, sem substituir as avaliações clínicas e comportamentais.

Além disso, ao relacionar a amplitude com a forma de apresentação do estímulo, observou-se que, na condição binaural, as crianças apresentaram maiores respostas de amplitude, sugerindo um efeito de somação binaural. Esse achado indica que a estimulação simultânea em ambas as orelhas pode favorecer a identificação da resposta neural, possivelmente devido à integração sincrônica dos sinais auditivos ao longo da via auditiva^(10,24)^.

O efeito da condição de estimulação foi altamente significativo, indicando que a apresentação binaural influencia na amplitude das respostas. Além disso, a interação entre grupo e condição não foi significativa, o que demonstra que a somação binaural exerce um efeito positivo na amplificação da resposta neural, independentemente do grupo. A ausência de diferenças entre os lados na condição monoaural sugere que a estimulação unilateral gera respostas semelhantes, independentemente da orelha estimulada. Esses achados podem justificar a opção, adotada em alguns estudos com potenciais evocados auditivos, pela apresentação do estímulo em condição monoaural — seja de forma alternada em ambas as orelhas ou exclusivamente na orelha direita^(2,7-9,25)^.

A preferência pela orelha direita nas apresentações monoaurais fundamenta-se na dominância hemisférica esquerda para o processamento da linguagem. Considerando que as informações auditivas percorrem predominantemente vias cruzadas no sistema nervoso central, os estímulos linguísticos apresentados na orelha direita são direcionados ao hemisfério esquerdo, favorecendo uma codificação neural mais rápida e eficiente dos sons da fala^(11,26)^.

A ausência de efeito significativo da condição de estimulação (monoaural ou binaural) sobre a latência sugere que a forma de apresentação do estímulo não interfere substancialmente no processamento dos aspectos temporais da fala. Isso indica que, independentemente da orelha testada ou da estimulação simultânea, a integridade das respostas neurais em crianças com transtorno fonológico pode permanecer alterada. Conforme apontado^(11)^, embora o FFR seja um potencial evocado auditivo sensível à entrada sensorial — isto é, à forma como o som chega ao sistema auditivo, a apresentação do estímulo em ambas as orelhas pode otimizar a robustez da resposta neural, mas não é suficiente para compensar déficits específicos no processamento e codificação auditiva em crianças com transtorno fonológico.

### Implicações para a pesquisa e limitações do estudo

Os resultados obtidos neste estudo indicam que o FFR pode ser um marcador objetivo na investigação das alterações na codificação auditiva em crianças com transtorno fonológico, especialmente nas características transitórias do som, evidenciadas pelo aumento das latências dos componentes. No entanto, novas pesquisas podem aprofundar a análise do FFR, tanto no domínio do tempo quanto da frequência, em crianças com transtorno fonológico e outros TSF, a fim de comparar as características das respostas auditivas nos diferentes tipos desses transtornos. Isso pode fornecer uma melhor compreensão das semelhanças e discrepâncias no processamento auditivo em crianças com TSF, contribuindo assim para o aprimoramento das estratégias de intervenção terapêutica nesses transtornos.

Sugere-se a realização de estudos longitudinais para acompanhar a maturação da via auditiva e, principalmente, para avaliar os avanços na codificação das informações auditivas com as intervenções fonoaudiológicas. Isso permitiria investigar se os progressos nas habilidades linguísticas e auditivas de crianças com transtorno fonológico estão associados à normalização das respostas do FFR, o que poderia servir como um marcador objetivo da eficácia terapêutica, além de auxiliar na definição dos objetivos terapêuticos a serem estimulados.

Além disso, novos estudos podem correlacionar a gravidade do transtorno fonológico e a quantidade de processos fonológicos com os achados de latência e amplitude, investigando se crianças com maior comprometimento fonológico e maior gravidade apresentam respostas do FFR mais alteradas, assim como associar os achados eletrofisiológicos do FFR a dados comportamentais obtidos por meio de testes que avaliam o processamento auditivo central.

É importante considerar que fatores socioeconômicos e ambientais, como nível de escolaridade materna, renda familiar e tipo de escola, exercem influência significativa sobre o desenvolvimento auditivo e linguístico infantil. Estudos apontam que ambientes com menor estimulação linguística e maior vulnerabilidade social podem impactar negativamente o desenvolvimento das habilidades auditivas e de linguagem, independentemente da presença de alterações neurofisiológicas específicas^(27)^.

A ausência de controle e pareamento dessas variáveis entre os grupos representa uma limitação do presente estudo e deve ser considerada na interpretação dos resultados, uma vez que tais fatores podem atuar como variáveis intervenientes no desempenho auditivo e linguístico das crianças.

## CONCLUSÃO

Os resultados deste estudo indicaram que crianças com Transtorno Fonológico apresentam comprometimento na codificação neural dos sons da fala, evidenciado pelo aumento das latências — significativamente maiores na onda V do FFR — em comparação com crianças com desenvolvimento típico, independentemente da condição de estimulação. Adicionalmente, observou-se que a estimulação binaural promoveu latências menores e amplitudes maiores em relação às condições monoaurais, sugerindo um efeito de somação binaural.
